# Summer Biomass Variability and Spatial Interactions between European Sprat (*Sprattus sprattus*) and Moon Jellyfish (*Aurelia aurita*) in the Western Part of the Black Sea

**DOI:** 10.3390/ani13233691

**Published:** 2023-11-29

**Authors:** Vesselina Mihneva, Violin Raykov, Dimitar Petkov Dimitrov

**Affiliations:** 1 Institute of Fish Resources, Agricultural Academy, Bul. Primorski 4, 9000 Varna, Bulgaria; 2 Institute of Oceanology, Bulgarian Academy of Sciences, Str Parvi May 40, 9000 Varna, Bulgaria; vraykov@io-bas.bg (V.R.); dimpetdim@io-bas.bg (D.P.D.)

**Keywords:** small pelagic fish, gelatinous zooplankton, biomass estimation, spatial distribution

## Abstract

**Simple Summary:**

Between 2019 and 2022, scientific pelagic trawl surveys in the Western Black Sea monitored the biomass of sprat and moon jellyfish and their spatial variability in the summer. Investigations into the correlation patterns between the two plankton-feeding species revealed that gelatinous plankton can have a weak-to-moderate effect on the spatial distribution of sprat assemblages in coastal areas.

**Abstract:**

Over the past few decades, various causal connections between commercial small pelagic fish species and gelatinous zooplankton have been reported in the Black Sea, which affect the pelagic ecosystem. Recently, moon jellyfish regained dominance among gelatinous plankton; however, biomass fluctuations and interactions with small pelagic fish remain poorly understood. During the summers of 2019–2022, scientific pelagic trawl surveys in the Western Black Sea enabled simultaneous monitoring of small pelagic fish biomass, with sprat as the key species and moon jellyfish as an incidental catch. In total, 153 trawl hauls were conducted across four depth strata from 15 to 100 m, and a “swept area” method was used for biomass estimation. The sprat stock biomass ranged from 10,698 to 29,177 t, with an average value of 19,432 ± 4834 t. The total biomass of moon jellyfish was 2002 ± 868.73 t, and dense aggregations were observed in the coastal waters during certain years. Two scenarios of spatial interactions between planktivorous species were identified and linked to the formation of *A. aurita* aggregations. We found that changes in jellyfish density were associated with weak-to-moderate effects on the spatial distribution of sprat assemblages in coastal areas.

## 1. Introduction

Small pelagic fish and gelatinous plankton (Ctenophora and Scyphozoa) are intermediate-level consumers in marine food webs. In many instances, plankton production that drives small pelagic fish also influences gelatinous zooplankton production, and these organisms frequently overlap in space, time, and diet in coastal ecosystems [1]. However, numerous studies have revealed complex interactions between these two groups of predators, ranging from commensalism and improved survival of juvenile fish to mostly negative impacts on fish, owing to competition for shared food sources and predation during the early life stages of fish [2,3,4,5]. Climate events, overfishing, eutrophication, translocation, and habitat changes can affect the long-term relationships between small pelagic fish and gelatinous zooplankton [6,7,8,9]. Intraguild interactions within the gelatinous species faction, such as competition for food, predator–prey relationships, and predation of larval stages, impact their diversity and abundance [10,11,12,13] and may introduce variability to the connections between small pelagic fish and gelatinous plankton.

The Black Sea ecosystem is susceptible to multiple stressors, including overfishing, eutrophication, chemical contamination, and an increase in the number of introduced species [14,15,16,17]. This semi-enclosed brackish anoxic sea is also characterized by low species diversity. Although small pelagics are the focus of fisheries, only a few species are widespread, including European anchovy (*Engraulis encrasicolus*), European sprat (*Sprattus sprattus*), whiting (*Merlangius merlangus*), and Mediterranean horse mackerel (*Trachurus mediterraneus*) [18]. While anchovy generates the highest total biomass and catch in the Black Sea, it is mainly concentrated in the southern and eastern regions [19], whereas sprat is a significant commercial species in the western and northern regions. Of the five gelatinous species commonly found in the Black Sea, three were native to the area (*Aurelia aurita*, *Rhizostoma pulmo*, and *Pleurobrachia pileus*), whereas two non-native ctenophores (*Mnemiopsis leidyi* and *Beroe ovata*) were introduced in the late 1980s and the 1990s [20,21].

Overfishing is believed to trigger trophic cascades and regime shifts in the Black Sea food web, with detrimental consequences for the aquatic environment [22,23,24]. Long-term data suggest that the proportion of gelatinous plankton among the total resources of planktivorous predators has increased [25], likely because of the massive development of the scyphomedusae *A. aurita* in the 1980s [26,27] and the subsequent “explosion” of the invasive ctenophore *M. leidyi* in the 1990s [28,29]. The spread of this new species is consistent with a decrease in fodder zooplankton and small pelagic fish stocks [30,31,32,33]. During this period, the ctenophore *M. leidyi* developed an antagonistic relationship with the native jellyfish *A. aurita* because of competition for food and predation of juveniles [34,35]. Following the introduction of the second new ctenophore, *B. ovata*, which feeds on other ctenophores, and the establishment of a predator–prey relationship between *B. ovata* and *M. leidyi*, the biomass of *M. leidyi* began to decline [36,37,38]. Small pelagic fish stocks have shown signs of recovery; in particular, during the late 1990s and the 2000s, catches and biomass began to increase [39]. The proportion of moon jellyfish has increased and currently has the highest biomass among gelatinous species [40,41,42,43].

Seasonal fluctuations in *A. aurita* population dynamics have been documented in the Black Sea, with biomass usually peaking during the spring and summer months [44,45]. Despite significant regional and seasonal variability, the amount of this species tends to increase near the shore. Spatially, sprat and moon jellyfish may overlap, as sprat migrates between foraging grounds in coastal waters and offshore spawning grounds [46], and feeding intensifies in spring [47] in parallel with the increased quantities of moon jellyfish in coastal waters. Both species are zooplanktivorous predators [48,49,50,51]; but their dietary overlap has not been completely explored. Observations in the North-western Black Sea suggest that moon jellyfish development can impede the formation of sprat agglomerations during the spring and summer [52,53]. However, the role of jellyfish in sprat distribution has not been fully elucidated. Specifically, the stock biomass of *A. aurita* and their relationship with commercial small pelagics in the Western Black Sea remain largely unexplored.

In recent years, extensive pelagic trawl surveys have been conducted in Bulgarian Black Sea waters, enabling the monitoring of the variability of small pelagic fish and the less-examined gelatinous plankton in bycatch. In terms of pelagic surveys, our study goals included: (i) estimating the biomass of sprat and moon jellyfish in the Western Black Sea during the summers of 2019–2022; (ii) examining their spatial distribution by depth strata; and (iii) testing patterns of spatial interactions between moon jellyfish and sprat.

## 2. Materials and Methods

**Figure 1:**
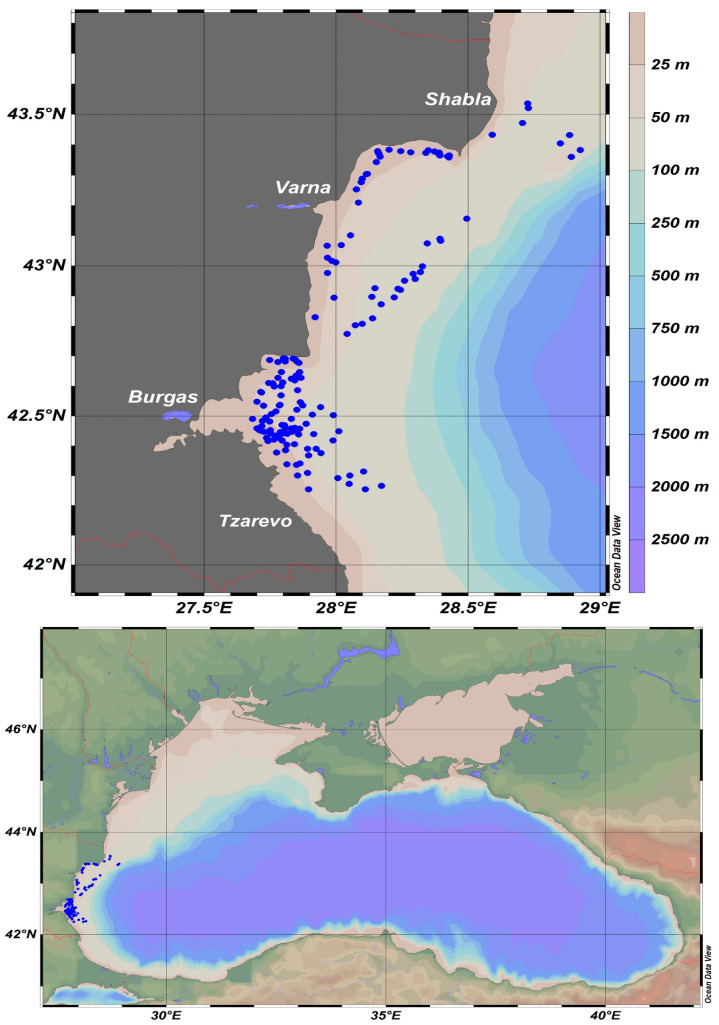
Map of pelagic trawling locations in the Bulgarian sector of the Black Sea during 2019–2022.

The data in this study were acquired from scientific pelagic trawl surveys performed in the Bulgarian Sector of the Black Sea during the summer months of 2019–2022, executed within the framework of the National Fisheries Data Collection Program, focusing on the biomass assessment of small pelagic fish species (mainly sprat) [54], and the moon jellyfish, which was collected as a bycatch. The surveys encompassed the territorial waters between cape Shabla (north) and Tzarevo (south) within the 100 m isobath (Figure 1).

One fishing vessel (with a length of 24.53 m, and tonnage of 142 GT) was employed for all surveys, and a standard pelagic trawl was applied (trawl vertical opening of 7 m, effective part of 13.5 m, mesh size of 7 × 7 mm).

Sampling was conducted in 40 randomly chosen fields, each of which was a rectangle with sides of 5′Lat × 5′Long, with a total area of 62.58 km^2^. The survey region was divided into four strata depending on depth: stratum 1 (<25 m), stratum 2 (25–50 m), stratum 3 (50–75 m), and stratum 4 (75–100 m).

Between 37 and 41 seasonal pelagic trawls were performed (Table 1), and the duration of each haul was 30–40 min at an average trawling speed of 2.7 knots. Trawling was performed only during the light part of the day, when both species were common in the deeper layers down to the thermocline zone. Before trawling, the depth of the thermocline was measured using an echo sounder SIMRAD NSO evo3 to determine the optimal trawl position.

A total of 153 trawls were executed and the quantities of sprat catch, and jellyfish bycatch were measured onboard the fishing vessel.

The method of “swept area” [55] was applied to estimate the sprat and jellyfish biomass. For each trawl haul, the catch per unit area was calculated by dividing the catch by the swept area of the fishing gear, based on Equation (1):

CPUA = C/a,(1)

where C is the catch weight (kg), and a is the swept area of the fishing gear (in km^2^).

The swept area was calculated using Equation (2):

a = d·h·X2,(2)

where d is the distance covered by the trawl (m), h is the length of the head rope (m), and X2 is the wing spread as a fraction of the head rope length (usually between 0.4 and 0.6).

To estimate the distance covered by the trawl, the GPS coordinates were first transformed into UTM coordinates (zone 35), as UTM coordinates are based on a two-dimensional Cartesian system that uses meters as a unit. Then the Pythagorean theorem was used to find the distance between the Cartesian coordinates from Equation (3):

d = ((UTM (35) _Latitude1_ − UTM (35) _Latitude2_)^2^ + (UTM (35) _Longitude1_ − UTM (35) _Longitude2_)^2^)^0.5^ (3)

where UTM (35) _Latitude1/Logitude1_ and UTM (35) _Latitude2/Longitude2_ were the initial and end coordinates of the trawling.

The biomass (B) is estimated using Equation (4):

B = CPUA/q,(4)

where CPUA is the catch per unit area and q is the catchability coefficient. The value of q varies between 0.5 and 1.0. In the Black Sea, the catchability coefficient for small pelagic fish is q = 1 [54].

The total biomass (t) of the two species in the fishing area was determined by multiplying the calculated average biomass (t·km^−2^) by the fishing ground area (km^2^), which was estimated using a geographic information system.

Ocean Data View software (ODV 5.6.3, https://odv.awi.de, 2023) was used to visualize the spatial distribution of the biomass of the two species in the Western Black Sea.

Statistical methods included analysis of variance (ANOVA) and correlation analysis based on the Pearson coefficient (performed in XLSTAT 19.03). To test the significance of the inter-annual variation in biomass, we used ANOVA. The initial datasets were normalized with a square root transformation to compress the high values and spread out the low values. The outliers were checked using Tukey’s fences. Based on this test, 5.9% of the sprat data were removed as outliers, and normality tests were performed using the Shapiro–Wilk and Jarque–Bera tests and related Q–Q plots. To determine whether the data had equal or similar variances across different groups of samples (i.e., homogeneous distribution), plots of predicted values versus standardized residuals were used. Considering the main assumptions and the applied ANOVA, the year-to-year variability of the sprat biomass was statistically significant.

The Pearson correlation method was used to estimate the degree of correlation between jellyfish and sprat biomass and to test the patterns of spatial interactions between both species, with inclusion of the “depth” factor in the analysis. The Pearson correlation coefficient is sensitive to outliers and the distribution of data. Thus, the biomass datasets of both species were square root transformed and tested for outliers and normality before analysis.

## 3. Results

### 3.1. Variability of Biomass of Sprat and Moon Jellyfish

The average value of sprat biomass for the observed summers of 2019–2022 was 2.394 t·km^−2^ ± 0.207 SE, which was significantly higher than the average moon jellyfish biomass of approximately 0.243 t·km^−2^ ± 0.047 SE (Table 2).

**Figure 2:**
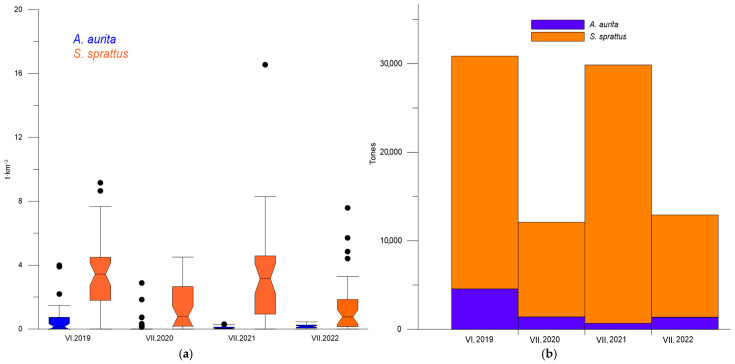
Box plot: biomass (t·km^−2^) of *S. sprattus* and *A. aurita* (**a**); stock biomass (t) of *S. sprattus* and *A. aurita* in the fishing area during the summers of 2019–2022 (**b**).

A noticeable fluctuation in sprat biomass was detected, with distinct “high” and “low” summer levels. The average sprat biomass ranged between 3.28 and 3.64 t·km^−2^ in 2019–2021, but decreased by almost 60% in 2020 and 2022. Concurrently, the stock biomass of sprat in the fishing area oscillated between 26,298 and 29,177 t during the “high biomass” summers and dropped to 10,689–11,553 t in the “low biomass” summers (Figure 2b).

The total quantity of *A. aurita* varied from 689 to 4562 t, with an average level of 2001.75 t ± 868.73 SE. The moon jellyfish biomass peaked in the summer of 2019, but decreased in the following period, with a minimum in 2021 (Table 2). The maximum sprat biomass was observed in the summer of 2021, paralleling the lowest biomass of *A. aurita*. Additionally, the percentage differences between the average summer minimal and maximal biomass of the sprat were 92.36% and 147.56%, respectively, for moon jellyfish, indicating high variability.

ANOVA revealed statistically significant interannual variations in summer sprat biomass (*p* < 0.0001) (Table 3).

**Figure 3:**
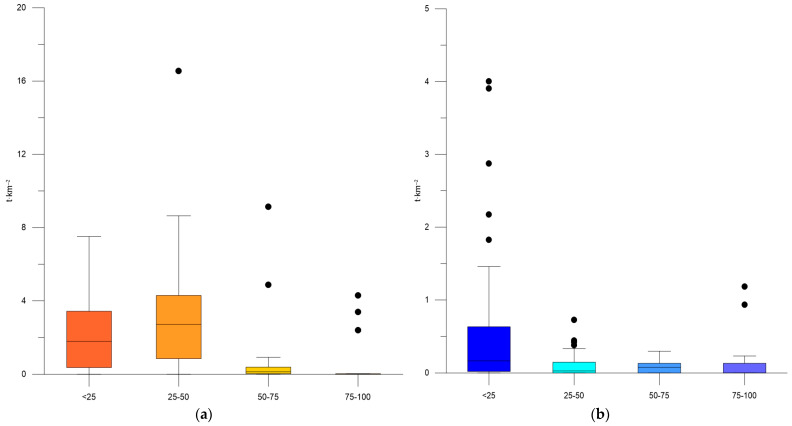
Box plots: biomass (t·km^−2^) of *S. sprattus* (**a**) and *A. aurita* (**b**) by depth strata, aggregated summer data for 2019–2022.

Considering the depth distribution of biomass, it was found that the sprat biomass increased up to maximal levels of 7.51–16.55 t·km^−2^ at depths below 50 m (Table 4, Figure 3a). Additionally, the biomass of moon jellyfish reached a maximum of 2.88–4.004 t·km^−2^ in coastal waters, whereas lower concentrations were observed in deeper waters (Table 4, Figure 3b).

### 3.2. Spatial Dynamics of S. sprattus and A. aurita

**Figure 4:**
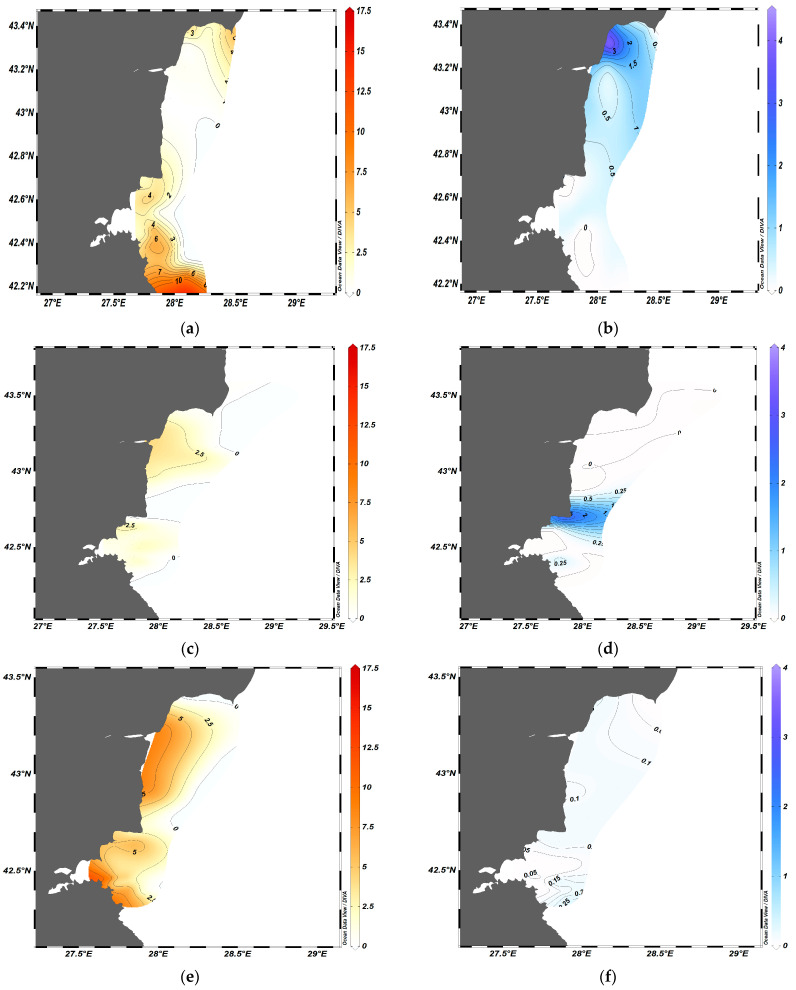

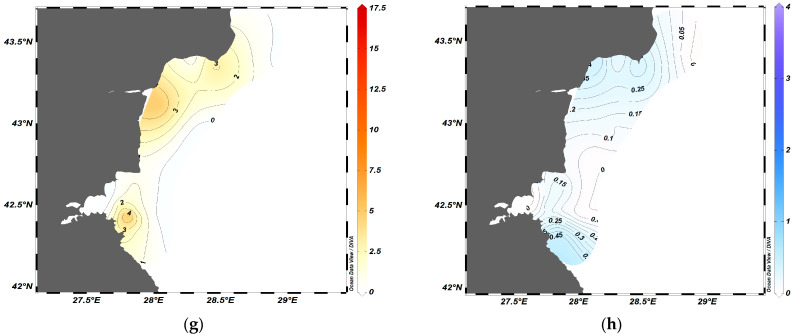
Spatial distribution of biomass (t·km^−2^) of sprat (**a**,**c**,**e**,**g**) and moon jellyfish (**b**,**d**,**f**,**h**) during the summer months of 2019–2022.

ODV mapping allowed the visualization of the spatial distribution of the biomass of the two species (Figure 4), and showed that usually two to three sprat agglomerations with a biomass of 4–5 t·km^−2^ could be registered within the wide shelf area in summer (Figure 4a,c,e,g). *A. aurita* displayed a patchy distribution and swarm formation with a maximum biomass of 3–4 t·km^−2^ during the summers of 2019–2020 (Figure 4b,d). During this period, low sprat biomass was detected in regions with high concentrations of jellyfish, indicating the possibility of spatial avoidance between the two species (Figure 4a,c).

Conversely, during the summers of 2021 and 2022, the moon jellyfish was dispersed along the coast with generally low biomass, and the maximal concentration did not exceed 0.5 t·km^−2^ (Figure 4f,h). Sprat formed large dense agglomerations in the summer of 2021 (Figure 4).

Taking into account the variability in jellyfish biomass and swarm formation, we identified two main scenarios of spatial distribution: the presence of dense jellyfish aggregations during the summers of 2019–2020, and low biomass during the summers of 2021–2022.

### 3.3. Correlation Analysis

The significance and strength of the relationships between sprat and moon jellyfish biomass were tested using a Pearson correlation coefficient, under the conditions of the two selected scenarios: (1) formation of moon jellyfish patches with biomass of 3–4 t·km^−2^; (2) low jellyfish biomass (<0.5 t·km^−2^) and lack of swarms (Table 5). In addition, the depth of the observations was included in the analysis.

In the first scenario, the analysis indicated a linear relationship (*p* = 0.009) between the biomass of both species. In this case, the association between moon jellyfish and sprat biomass was explained by a weak negative correlation (r = −0.301, Table 5(1)).

The scenario, conducted with data from the summers of 2021–2022, showed a lack of correlation between species (Table 5(2)). In this case, a moderately negative relationship between sprat biomass and depth was found (r = −0.622, *p* = 0.0001).

In both scenarios, the analysis indicated a weak negative linear relationship between *A. aurita* and depth, whereas sprat correlated moderately negatively with depth only in the second scenario.

It can be inferred that changes in jellyfish density were associated with weak-to-moderate effects on the distribution of sprat in the study area. Although a weak negative linear relationship was observed between the two species in the first scenario, the second scenario showed an indirect moderate effect on the depth orientation of the sprat. The results from the two scenarios allowed us to conclude that the direction and strength of the linear correlation between sprat biomass and depth can change depending on jellyfish density.

## 4. Discussion

Many studies have examined the consequences of gelatinous plankton development on pelagic communities, ranging from temporary alterations in community structure to long-term effects on food webs and trophic cascades [56,57,58,59,60,61,62,63]. Jellyfish have been observed to cause both fish-killing events and chronic gill damage in marine-farmed fish [64,65]. Furthermore, there is evidence that the diet, recruitment, and stock dynamics of commercially important small pelagic fish can be affected by “blooms” of gelatinous plankton, which share the same food sources [66,67]. However, few studies have explored the spatial relationships between gelatinous plankton and small pelagics [68,69,70], and such data are scarce in the Black Sea [71].

Sprat is the second most abundant species of small pelagic fish in the Black Sea, with the highest biomass in the 1970s and 1980s, followed by a sharp decline in the early 1990s, and some recovery in subsequent years [72,73]. The sprat population exhibited a distinct cyclical pattern, with years of high recruitment followed by years of low-to-moderate recruitment and corresponding fluctuations in spawning stock biomass [74]. Previous studies in the Western Black Sea showed that the population of sprat ranged from 32,000 to 75,000 t in the late spring and summer of 2007–2010 [49]. Our study confirmed a statistically significant interannual variability in summer sprat biomass, and found the highest stock biomass of 26,298 t and 29,177 t in 2019 and 2021, respectively, with a decrease of almost 60% in 2020 and 2022. The data from this study indicated a decreasing trend in the summer biomass of sprat compared with previously published data for the region. These results, along with the evidence of decreasing sprat population parameters, such as the size and age structure of the catch and absolute individual fecundity [75,76], suggest that stock biomass has not fully recovered over time.

Understanding the spatial distribution of commercial fish stocks is a key factor for effective fishery management [77]. Sprat spatial distribution can be influenced by various factors such as environmental conditions, plankton productivity, food availability, predation and competition, and fishing pressure. The spatial structure and distribution of sprat and their interactions in the Black Sea have not been well studied. Recently, predictive spatial distribution models of small pelagics have been developed for the Western Black Sea using biological interactions (mesozooplankton) and abiotic factors, such as temperature, salinity, dissolved oxygen, and current velocity [78]. Predictors such as mesozooplankton biomass and salinity appear to be important factors in assessing sprat habitat suitability in the Western Black Sea. The impact of gelatinous plankton blooms on the spatial structure of small pelagic fish in this region has not yet been explored, mostly because of the lack of simultaneous data collection for both groups. Thus, the current surveys, which provide an estimate of the biomass and distribution of the two species in the pelagic zone, are a step towards a more comprehensive spatial analysis.

Moon jellyfish, which is currently the predominant gelatinous plankton species in the Black Sea, displays significant biomass variation in the region [52,53]. This species has a complex life cycle involving free-swimming jellyfish and benthic polyps, which explains the high level of variability in biomass [79]. Rocky shelf areas create a favorable environment for jellyfish development, and the influx of nutrients from large rivers supports the growth of phytoplankton and zooplankton, which could potentially affect the biomass of higher-level consumers in the food web [52,53]. In the Bulgarian Black Sea sector, the total jellyfish biomass varied from 689 to 4563 t, with a maximum level in 2019, whereas the total biomass was below 1400 t in most summers. Therefore, the average biomass of *A. aurita* was approximately one-tenth that of sprat. Comparing the spatial distribution of the two species, two to three main agglomerations of sprat were observed, distributed within a wide shelf, with biomasses of 4–5 t·km^−2^. We considered a jellyfish biomass threshold of 3–4 t·km^−2^ as an indicator of swarm presence. Two scenarios were outlined, with data from the summers of 2019–2020, which had elevated levels of jellyfish compared to the subsequent period of summers 2021–2022, where the jellyfish biomass was low. A weak linear negative relationship between the biomass of sprat and jellyfish only emerged in the presence of jellyfish aggregations, but the two species were uncorrelated at low jellyfish biomass. Furthermore, the pattern of linear relationships between the sprat biomass and depth could change under different scenarios. A moderately negative relationship between sprat distribution and depth was observed at low jellyfish densities. Although the Pearson correlation method is effective in outlining the patterns and strength of linear relationships, it has limitations in explaining the underlying mechanisms. Further research is needed to better understand the roles of environmental factors, diet, and presence of other competitors or predators in shaping the observed patterns of species dynamics.

Finally, changes in mesozooplankton communities in the Black Sea, linked to the introduction and rapid proliferation of a new cyclopoid copepod species, *Oithona davisae*, after the 2000s [80,81,82,83], should be considered, as they could potentially support moon jellyfish populations. Investigations in the native habitats of *O. davisae* showed that this copepod dominated *A. aurita* food, and jellyfish prosperity was related to the high abundance of *O. davisae* populations [84]. Studies on sprat feeding in the Black Sea have indicated that large copepods, such as *Calanus euxinus* and *Pseudocalanus elongatus*, as well as chaetognaths, are the main food sources, but Oithonidae contribute to a small portion of the diet [49,85,86]. It is possible that changes in mesozooplankton species composition and shifts in the trophic web can influence gelatinous plankton communities and add variability to sprat population distribution. Dietary overlap and food competition between sprat and moon jellyfish have not been well studied in the Black Sea, and further research is required to understand the ecological interactions between these species and their potential impact on marine ecosystems and fisheries. Simultaneous monitoring of commercial fish and gelatinous plankton populations can help detect changes in ecosystem structure or function, and may be useful for making ecosystem-based management decisions [87,88].

## 5. Conclusions

This study focused on estimating the biomass and spatial distribution of sprat and moon jellyfish, which are key plankton-feeding species in the Western Black Sea. The summer pelagic trawl surveys revealed significant interannual variability in sprat biomass, with a decreasing trend compared to previous studies. The average biomass of *A. aurita* was estimated to be approximately one-tenth of that of the sprat. However, dense aggregations with biomass comparable to that of sprat have been observed in coastal areas over certain years. Two scenarios of spatial interaction between sprat and jellyfish were identified, induced by the formation of *A. aurita* swarms. Linear patterns of relationships were found between the variables in the two scenarios, and changes in jellyfish density were associated with weak-to-moderate effects on sprat biomass distribution in coastal waters.

## Figures and Tables

**Table 1:** Distribution of sampling trawls per study period and depth stratum. (The percentage share of collected samples was calculated separately for each variable).

Variable	Categories	Trawl Counts	%
year	VI.2019	37	24.183
	VII.2020	38	24.837
	VII.2021	41	26.797
	VII.2022	37	24.183
depth stratum	1 (<25 m)	43	28.105
	2 (25–50 m)	81	52.941
	3 (50–75 m)	16	10.458
	4 (75–100 m)	13	8.497

**Table 2:** Average biomass (t·km^−2^) of *S. sprattus* and *A. aurita* (with standard error, SE) and stock biomass (t) estimated for the summer months of 2019–2022 in the Western Black Sea.

Species	Average Biomass t·km^−2^	SE	Stock Biomass
VI.2019			
*S. sprattus* *A. aurita*	3.283	0.414	26,298
	0.57	0.161	4562
VII.2020			
*S. sprattus* *A. aurita*	1.336	0.221	10,698
	0.174	0.092	1396
VII.2021			
*S. sprattus* *A. aurita*	3.64	0.52	29,177
	0.086	0.012	689
VII.2022			
*S. sprattus* *A. aurita*	1.442	0.294	11,553
	0.17	0.021	1361
Average of summers 2019–2022			
*S. sprattus*	2.394	0.207	19,431.69
*A. aurita*	0.243	0.047	2001.75

**Table 3:** ANOVA statistics of variations of sprat biomass (square root transformed data) for the observed summer period (2019–2022) in the Western Black Sea.

Source of Variation	SS	df	MS	F	*p*-Value	F Crit
Between Groups	24.735	3	8.245	20.486	7.57 × 10^−11^	2.678
Within Groups	49.907	124	0.403			
Total		127				

**Table 4:** Biomass (t·km^−2^) of sprat and moon jellyfish by depth strata during the summer months of 2019–2022.

Year	Depth Stratum	*S. sprattus* Biomass (t·km^−2^) ± SD	Min–Max Biomass of *S. sprattus*	*A. aurita* Biomass (t·km^−2^) ± SD	Min–Max Biomass of *A. aurita*
VI.2019	<25	1.991 ± 1.630	0–4.498	1.217 ± 1.329	0–4.004
	25–50	4.428 ± 2.205	0–8.644	0.086 ± 0.148	0–0.435
	50–75	7.008 ± 3.029	4.866–9.15	0.076 ± 0.108	0–1.152
	75–100	2.150 ± 1.075	0–4.30	0.605 ± 0.541	0.064–1.186
VII.2020	<25	1.419 ± 1.469	0–3.523	0.489 ± 1.014	0.002–2.877
	25–50	1.327 ± 1.299	0–4.509	0.071 ± 0.171	0–0.729
	50–75	0.076 ± 0.092	0–0.179	0	0
	75–100	1.933 ± 1.767	0–3.400	0	0
VII.2021	<25	3.045 ± 3.175	0.01–7.509	0.103 ± 0.052	0.02–0.147
	25–50	4.322 ± 3.242	0.508–16.551	0.076 ± 0.088	0–0.303
	50–75	0.388 ± 0.465	0.118–0.926	0.124 ± 0.097	0.118–0.132
	75–100	0.0002 ± 0.0003	0–0.001	0.032 ± 0.056	0–0.097
VII.2022	<25	2.013 ± 1.552	0.379–4.85	0.238 ± 0.101	0.095–0.411
	25–50	2.012 ± 2.111	0.104–7.592	0.172 ± 0.133	0.029–0.445
	50–75	0.127 ± 0.137	0–0.394	0.119 ± 0.114	0–0.299
	75–100	0.011 ± 0.019	0–0.033	0.044 ± 0.077	0–0.133
Average for 2019–2022	<25	2.056 ± 1.954	0–7.509	0.590 ± 0.99	0–4.004
	25–50	3.103 ± 2.753	0–16.551	0.095 ± 0.138	0–0.729
	50–75	1.026 ± 2.473	0–9.150	0.092 ± 0.096	0–0.299
	75–100	0.779 ± 1.525	0–4.300	0.204 ± 0.391	0–1.186

**Table 5:** Correlation matrix (with *p*-values) between the square root transformed biomasses of moon jellyfish, sprat, and depth (bold indicates statistically significant values of *p* < 0.05).

1. Scenario 1: Occurrence of *A. aurita* aggregations with biomass of 3–4 t·km^−2^ (2019–2020).		
**Variables**	***A. aurita* Biomass**	***S. sprattus* Biomass**
***A. aurita* biomass**	1	
***S. sprattus* biomass**	**−0.301 (*p* = 0.009)**	1
**Depth**	**−0.253 (*p* = 0.028)**	−0.12 (*p* = 0.305)
2. Scenario 2: Scattered presence of *A. aurita* by generally low jellyfish biomass of < 0.4 t·km^−2^ (2021–2022).		
**Variables**	***A. aurita* Biomass**	***S. sprattus* Biomass**
***A. aurita* biomass**	1	
***S. sprattus* biomass**	0.168 (*p* = 0.165)	1
**Depth**	**−0.362 (*p* = 0.002)**	**−0.622 (*p* < 0.0001)**

## Data Availability

The data presented in this study are available upon request from the corresponding author. The data are not publicly available due to institutional restrictions.

## References

[1] Robinson K.L., Ruzicka J.J., Decker M.B., Brodeur R.D., Hernandez F.J., Quiñones J., Acha E.M., Uye S.-I., Mianzan H., Graham W.M. (2014). Jellyfish, forage fish, and the world’s major fisheries. Oceanography.

[2] Purcell J.E., Arai M.N. (2001). Interactions of pelagic cnidarians and ctenophores with fish: A review. Hydrobiologia.

[3] Brodeur R.D., Suchman C.L., Reese D.C., Miller T.W., Daly E.A. (2008). Spatial overlap and trophic interactions between pelagic fish and large jellyfish in the northern California Current. Mar. Biol..

[4] D’Ambra I., Graham W.M., Carmichael R.H., Hernandez F.J. (2015). Fish rely on scyphozoan hosts as a primary food source: Evidence from stable isotope analysis. Mar. Biol..

[5] Tilves U., Sabatés A., Blázquez M., Raya V., Fuentes V. (2018). Associations between fish and jellyfish in the NW Mediterranean. Mar. Biol..

[6] Parsons T.R., Lalli C.M. (2002). Jellyfish population explosions: Revisiting a hypothesis of possible causes. La Mer.

[7] Lynam C.P., Gibbons M.J., Axelsen B.E., Sparks C.A.J., Coetzee J., Heywood B.G., Brierley A.S. (2006). Jellyfish overtake fish in a heavily fished ecosystem. Curr. Biol..

[8] Richardson A.J., Bakun A., Hays G.C., Gibbons M.J. (2009). The jellyfish joyride: Causes, consequences, and management responses to a more gelatinous future. Trends Ecol. Evol..

[9] Robinson K.L., Graham W.M. (2013). Long-term change in the abundances of northern Gulf of Mexico scyphomedusae *Chrysaora* sp. and Aurelia spp. with links to climate variability. Limnol. Oceanogr..

[10] Purcell J. (1991). A review of cnidarian and ctenophores feeding on competitors in the plankton. Hydrobiologia.

[11] Boero F. (2013). Review of Jellyfish Blooms in the Mediterranean and Black Sea.

[12] Shiganova T., Alekseenko E., Moskalenko L., Nival P. (2018). Modeling assessment of interactions in the black sea of the invasive ctenophores *Mnemiopsis leidyi* and *Beroe ovata*. Ecol. Model..

[13] Tang C., Sun S., Zhang F. (2020). Intraguild predation by polyps of three scyphozoan jellyfish: *Nemopilema nomurai*, *Aurelia coerulea*, and *Rhopilema esculentum*. J. Ocean. Limnol..

[14] (2007). TDA (2009) Transboundary Diagnostic Analysis (1996–2006).

[15] Shiganova T.A., Ozturk B., Briand F. (2010). Trend on increasing Mediterranean species arrival into the Black Sea. CIESM Workshop Monographs, No 39.

[16] Llope M., Daskalov G.M., Rouyer T.A., Mihneva V., Chan K., Grishin A.N., Stenseth N.C. (2011). Overfishing of top predators eroded the resilience of the Black Sea system, regardless of the climate and anthropogenic conditions. Glob. Change Biol..

[17] Oguz T. (2017). Controls of multiple stressors on black sea fishery. Mar. Sci..

[18] Shlyakhov V., Daskalov G., Oguz T. (2008). Marine living resources. State of the Environment of the Black Sea (2001–2006/7).

[19] FAO (2022). The State of Mediterranean and Black Sea Fisheries.

[20] Pereladov M.V. (1988). Some observations of changes in the biocenoses of the Sudak Bay of the Black Sea. Tez. III. Proceedings of the All-Union Conference in Marine Biology.

[21] Konsulov A., Kamburska L. (1998). Ecological determination of the new ctenophore—*Beroe ovata* invasion in the Black Sea. Okeanologia.

[22] Daskalov G.M., Grishin A., Rodionov S., Mihneva V. (2007). Trophic cascades triggered by overfishing reveal possible mechanisms of ecosystem regime shifts. Proc. Natl. Acad. Sci. USA.

[23] Oguz T., Akoglu E., Salihoglu B. (2012). Current state of overfishing and its regional differences in the Black Sea. Ocean Coast. Manag..

[24] Daskalov G., Boicenko L., Grishin A., Lazar L., Mihneva V., Shlyahov V., Zengin M. (2016). Architecture of collapse: Regime shift and recovery in a hierarchically structured marine ecosystem. Glob. Change Biol..

[25] Opdal A.F., Brodeur R.D., Cieciel K., Daskalov G.M., Mihneva V., Ruzicka J.J., Verheye H.M., Aksnes D.L. (2019). Unclear associations between small pelagic fish and jellyfish in several major marine ecosystems. Sci. Rep..

[26] Gomou M., Kuprianov A. (1980). Estimation of the abundance and distribution of the medusae *Aurelia aurita* in the eastern part of the Black Sea. Pelagic Ecosystem of the Black Sea.

[27] Zaicev Y.P., Polistuk L.N. (1984). An outbreak of the jellyfish *A. aurita* in the Black Sea. Sea Ecol..

[28] Vinogradov M.Y., Shushkina E.A., Musayeva E.I., Sorokin P.Y. (1989). A newly acclimatized species in the Black Sea: The ctenophore Mnemiopsis (Ctenophora: Lobata). Oceanology.

[29] Vinogradov M., Shushkina E. (1992). The temporal changes in the structure of the zoocenosis in open sea regions of the Black Sea. Oceanology.

[30] Shiganova T., Özsoy E., Mikaelyan A. (1997). Mnemiopsis leidyi abundance in the Black Sea and its impact on the pelagic community. Sensitivity to Change: Black Sea, Baltic Sea, and North Sea.

[31] Shiganova T. (1998). Invasion of the Black Sea by ctenophore *Mnemiopsis leidyi* and recent changes in pelagic community structure. Fish. Ocean..

[32] Niermann U., Dumont H., Shiganova T., Niermann U. (2004). Mnemiopsis leidyi: Distribution and Effect on the Black Sea Ecosystem during the First Years of Invasion in Comparison with Other Gelatinous Blooms. Aquatic Invasions in the Black, Caspian and Mediterranean Seas.

[33] Bilio M., Niermann U. (2004). Is the comb jelly really to blame for it all? *Mnemiopsis leidyi* and the ecological concerns about the Caspian Sea. Mar. Ecol. Prog. Ser..

[34] Mutlu E., Bingel F., Gucu A., Melnikov V., Niermann U., Ostr N., Zaika V. (1994). Distribution of the new invader *Mnemiopsis* sp. and the resident *A. aurita* and *Pleurobrachia pileus* populations in the Black Sea during 1991–1993. ICES J. Mar. Sci..

[35] Kovalev V., Piontkovski S. (1998). Interannual changes in the biomass of the Black Sea gelatinous zooplankton. J. Plankton Res..

[36] Kamburska L., Schrimpf W., Djavidni S., Shiganova T., Stefanova K. (2006). Addressing the Ecological Issue of the Invasive Species Special Focus on the Ctenophore Mnemiopsis Leidy (Agassiz, 1865) in the Black Sea.

[37] Vereshchaka A.L., Anokhina L.L., Lukasheva T.A., Lunina A.A. (2019). Long-term studies reveal major environmental factors driving zooplankton dynamics and periodicities in the Black Sea coastal zooplankton. PeerJ.

[38] Vereshchaka A.L., Shatravin A.V., Lunina A.A. (2022). Shifting seasonal timing of peak abundance of two invading ctenophore populations in the Black Sea during the period 1991–2017. ICES J. Mar. Sci..

[39] GFCM: Report of the Seventh Meeting of the Subregional Group on Stock Assessment in the Black Sea (SGSABS), 12–16 July 2021. https://www.fao.org/gfcm/technical-meetings/detail/en/c/1442355/.

[40] Anninsky B.E., Finenko G.A., Datsyk N.A., Gaevskaya A.V. (2016). The trophodynamic role of gelatinous predators in planktonic communities of the coastal regions of the Black Sea. Morskie biologicheskie issledovaniya: Dostizheniya i perspektivy: V 3–kh t.:sb. materialov Vseros. nauch. –prakt. konf. s mezhdunar. uchastiem, priuroch. k 145–letiyu Sevastopol’skoi biologicheskoi stantsii (Sevastopol, September 19–24, 2016/A.V.).

[41] Anninsky B.E., Ignatyev S.M., Finenko G.A., Datsyk N.A. (2019). Gelatinous macroplankton of the open pelagial and shelf of the Black Sea: Distribution in autumn 2016 and interannual changes in biomass and abundance. Mar. Biol. J..

[42] Harcota G., Bisinicu E., Tabarcea C., Țotiou A., Filimon A., Abaza V., Boicenco L., Timofte F. (2022). Distribution, and abundance of the macrozooplankton community in the Black Sea in 2021. Acad. Rom. Sci. Ann. Ser. Biol. Sci..

[43] Finenko G.A., Datsyk N.A., Anninsky B.E., Zagorodnyaya Y.A. (2022). Trophic relationships in the zooplankton–gelatinous zooplankton food chain in the shelf areas of the Crimean coast of the Black Sea. Mar. Biol. J..

[44] Mutlu E. (2001). Distribution and abundance of moon jellyfish (*Aurelia aurita*) and its zooplankton food in the Black Sea. Mar. Biol..

[45] Bat L., Satilimus H., Birinci-Ozdemir Z., Sahin F., Ustin F. (2009). Distribution and population dynamics of *Aurelia aurita* (Cnidaria; Scyphozoa) in the southern Black Sea. Northwestern J. Zool..

[46] Ivanov L., Beverton R.J.H. (1985). The fisheries resources of the Mediterranean. Part II: Black Sea. FAO Stud. Rev..

[47] Lipskaia N. (1960). Daily and seasonal feeding of Black Sea sprat (*Sprattus sprattus* phalericus Risso). Proc. Sevastopol Boil. Stn. RAN.

[48] Glushtenko T.I., Petrenko O.A. (2011). Feeding and estimation of the Black Sea sprat in 2009–2010. The Main Results of Complex Research in the Azov–Black Sea Basin and the World Ocean.

[49] Mihneva V., Raykov V., Grishin A., Stefanova K. Sprat feeding in front of the Bulgarian Black Sea coast. Proceedings of the 12th International Conference on the Mediterranean Coastal Environment, MEDCOAST 2015.

[50] Bișinicu E., Harcotă G.E., Țoțoiu A., Timofte F., Radu G. (2017). Romanian Black Sea Zooplankton and Its Role in the Diet of Sprattus in 2016–2017. Rev. Cercet. Mar. Rev. Rech. Mar. Mar. Res. J..

[51] Finenko G.A., Anninsky B.E., Datzyk N.A. (2021). Spatial Distribution, Population Structure, and Predatory Impact of Gelatinous Predators on Zooplankton Community in Inshore Waters off Crimean Coast of the Black Sea. J. Sib. Fed. Univ. Biol..

[52] Totoiu A., Galatchi M., Radu G. (2016). Dynamics of the Romanian sprat (*Sprattus sprattus*, Linnaeus 1758) fishery between evolution of the fishing effort and the state of the environmental conditions. Turk. J. Fish. Aquat. Sci..

[53] Țoțoiu A., Galațchi M., Danilov S., Radu G. (2017). Evolution of the Sprat (*Sprattus sprattus*, Linnaeus 1758) Population at the Romanian Littoral during 2008–2016. Cercet. Mar..

[54] Raykov V., Yankova M., Ivanova P., Mihneva V., Dimitrov D., Stefanova K., Stefanova E., Kotsev I., Dzembekova N., Zlateva I. (2020). Pelagic Trawl Surveys in the Bulgarian Marine Area 2017–2019.

[55] Sparre P., Venema S.C. (1998). Introduction to Fish Stock Assessment. Part 1: Manual.

[56] Behrends G., Schneider G. (1995). Impact of *A. aurita* medusae (Cnidaria, Scyphozoa) on the standing stock and community composition of mesozooplankton in Kiel Bight (western Baltic Sea). Mar. Ecol. Prog. Ser..

[57] Schneider B., Behrends G. (1998). Top–down control of a nertitic plankton system by *A. aurita* medusae: A summary. Ophelia.

[58] Daskalov G.M. (2002). Overfishing drives a trophic cascade in the Black Sea. Mar. Ecol. Prog. Ser..

[59] Møller L.F., Riisgård H.U. (2007). Impact of jellyfish and mussels on algal blooms caused by seasonal oxygen depletion and nutrient release from the sediment in a Danish fjord. J. Exp. Mar. Biol. Ecol..

[60] Malej A., Turk V., Lucic D., Benovic A. (2007). Direct and indirect trophic interactions of Aurelia sp. (Scyphozoa) in a stratified marine environment (Mljet Lakes, Adriatic Sea). Mar. Biol..

[61] Pitt K., Kingsford M., Rissik D., Koop K. (2007). Jellyfish modified the responses of planktonic assemblages to nutrient pulses. Mar. Ecol. Prog. Ser..

[62] Hosia A., Augustin C., Dinasquet J., Granhag L., Paulsen M., Riemann L., Rintala J., Setälä O., Talvitie J., Titelman J. (2015). Autumnal bottom-up and top-down impacts of *Cyanea capillata*: A mesocosm study. J. Plankton Res..

[63] Jaspers C., Acuña J., Brodeur R. (2015). Interactions of gelatinous zooplankton within marine food webs. J. Plankton Res..

[64] Baxter E.J., Sturt M.M., Ruane N.M., Doyle T.K., McAllen R., Harman L., Rodger H.D. (2011). Gill damage to Atlantic salmon (Salmon salar) caused by the common jellyfish (*Aurelia aurita*) under experimental challenge. PLoS ONE.

[65] Bosch-Belmar M., Milisenda G., Basso L., Doyle T.K., Leone A., Stefano Piraino S. (2021). Jellyfish Impacts on Marine Aquaculture and Fisheries. Rev. Fish. Sci. Aquac..

[66] Shiganova T.A., Bulgakova Y.V. (2000). Effects of gelatinous plankton on Black Sea and Sea of Azov fish and their food resources. ICES J. Mar. Sci..

[67] Oguz T., Salihoglu B., Fach B. (2008). A coupled plankton–anchovy population dynamics model assessing nonlinear controls of anchovy and gelatinous biomass in the Black Sea. Mar. Ecol. Prog. Ser..

[68] Haraldsson M., Tönnesson K., Tiselius P., Thingstad T.F., Aksnes D.L. (2012). Relationship between fish and jellyfish as a function of eutrophication and water clarity. Mar. Ecol. Prog. Ser..

[69] Flynn B.A., Richardson A.J., Brierley A.S., Boyer D.C., Axelsen B.E., Scott L., Moroff N.E., Kainge P.I., Tjizoo B.M., Gibbons M.J. (2012). Temporal and spatial patterns in the abundance of jellyfish in the northern Benguela upwelling ecosystem and their link to thwarted pelagic fishery recovery. Afr. J. Mar. Sci..

[70] Decker M.B., Robinson K.L., Dorj S., Cieciel K.D., Barceló C., Ruzicka J.J., Brodeur R.D. (2018). Jellyfish and forage fish spatial overlap on the eastern Bering Sea shelf during periods of high and low jellyfish biomass. Mar. Ecol. Prog. Ser..

[71] Radu G., Maximov V., Anton E., Cristea M., Tiganov G., Totoiu A., Spanu A. State of the Fishery Resources in the Romanian Marine Area. Proceedings of the 4th Biennial Black Sea Scientific Conference, “Black Sea—Challenges towards Good Environmental Status”.

[72] Prodanov K., Mikhailov K., Daskalov G., Maxim C., Chashchin A., Arkhipov A., Shlyakhov V., Ozdamar E. (1997). Environmental Management of Fish Resources in the Black Sea and Their Rational Exploitation.

[73] Daskalov G. (2003). Long-term changes in fish abundance and environmental indices in Black Sea. Mar. Ecol. Prog. Ser..

[74] Scientific, Technical and Economic Committee for Fisheries (STECF) 14-14: Black Sea Assessments. https://stecf.jrc.ec.europa.eu/reports/medbs/-/asset_publisher/y1bW/document/id/903145?inheritRedirect=false&redirect=https%3A%2F%2Fstecf.jrc.ec.europa.eu%2Freports%2Fmedbs%3Fp_p_id%3D101_INSTANCE_y1bW%26p_p_lifecycle%3D0%26p_p_state%3Dnormal%26p_p_mode%3Dview%26p_p_col_id%3Dcolumn-2%26p_p_col_pos%3D1%26p_p_col_count%3D2%26_101_INSTANCE_y1bW_cur%3D3%26_101_INSTANCE_y1bW_keywords%3D%26_101_INSTANCE_y1bW_advancedSearch%3Dfalse%26_101_INSTANCE_y1bW_delta%3D20%26p_r_p_564233524_resetCur%3Dfalse%26_101_INSTANCE_y1bW_andOperator%3Dtrue.

[75] Shlyakhov V.A., Shlyakhova O.V. (2011). Dynamics of trawl catch structure of Black Sea sprat on the Black Sea Ukrainian shelf and impact of natural factors and fishery on them. Trudy YugNIRO.

[76] Zuev G., Skuratovskaya E. (2022). Long-term dynamics of reproductive potential and fishing of European sprat *Sprattus sprattus* (Linnaeus, 1758) (Pisces: Clupeidae) in the Black Sea. Thalassas.

[77] Cadrin S.X. (2020). Defining spatial structure for fishery stock assessment. Fish. Res..

[78] Zlateva I., Raykov V., Slabakova V., Stefanova E., Stefanova K. (2022). Habitat suitability models of five keynote Bulgarian Black Sea fish species to specific abiotic and biotic factors. Oceanologia.

[79] Matveev I.V., Adonin L.S., Shaposhnikova T.G., Podgornaya O.I. (2012). *Aurelia aurita*—Cnidarian, with a prominent medusiod stage. J. Exp. Zool. B Mol. Dev. Evol..

[80] Zagorodnyaya Y. (2002). *Oithona brevicornis* in the Sevastopol Bay: Is it a single event or a new invader in the Black Sea fauna?. Morskoy Ekol. J. Mar. Ecol. J..

[81] Temnykh A., Nishida S. (2012). New record of the planktoniccopepod *Oithona davisae* Ferrari and Orsi in the Black Seawith notes on the identity of “*Oithona brevicornis*”. Aquat. Invasions.

[82] Mihneva V., Stefanova K. (2013). Non-native copepod *Oithona davisae* in Western Black Sea: Seasonal and annual abundance variability. Bioinvasion Rec..

[83] Gubanova A.D., Garbazey O.A., Popova E.V., Altukhov D.A., Mukhanov V.S. (2019). *Oithona davisae*: Naturalization in the Black Sea, interannual and seasonal dynamics, and effects on the structure of the planktonic copepod community. Oceanology.

[84] Ishii H., Tanaka F., Purcell J.E., Graham W.M., Dumont H.J. (2001). Food and feeding of *Aurelia aurita* in Tokyo Bay with an analysis of stomach contents and measurement of digestion times. Jellyfish Blooms: Ecological and Societal Importance.

[85] Bisinicu E., Totoiu A., Timofte F., Harcota G., Oprea L. (2020). Interrelations between the mesozooplankton community and Sprattus sprattus from the Romanian Black Sea Area. Sci. Pap. Ser. D Anim. Sci..

[86] Saglam H., Yıldız I. (2019). Temporal and ontogenetic variation in the diet of three small pe-lagic fish in the Black Sea of Turkey. Mar. Biodivers..

[87] Samhouri J.F., Levin P.S., Harvey C.J. (2009). Quantitative evaluation of marine ecosystem indicator performance using food web models. Ecosystems.

[88] Samhouri J.F., Levin P.S., Ainsworth C.H. (2010). Identifying thresholds for ecosystem–based management. PLoS ONE.

